# Antidiarrheal activity of 80 % methanol extract of the aerial part of *Ajuga remota Benth (Lamiaceae)* in mice

**DOI:** 10.1186/s12906-016-1277-8

**Published:** 2016-08-22

**Authors:** Teshager Yacob, Workineh Shibeshi, Teshome Nedi

**Affiliations:** Department of Pharmacology and Clinical Pharmacy, School of Pharmacy, Addis Ababa University, P.O.Box 1176, Addis Ababa, Ethiopia

**Keywords:** *Ajuga remota*, Antidiarrheal activity, Castor oil, Enteropooling

## Abstract

**Background:**

In the Ethiopian traditional medicine, the aerial part of *Ajuga remota Benth* is used in the treatment of diarrhea. There are different mechanisms by which *Ajuga remota* may have antidiarrheal effect. Some of the possible mechanisms are through its anthelmintic and antibacterial activity. The present study aimed to evaluate whether the antidiarrheal effect of the plant also include antimotility and antisecretory effect using 80 % methanol extract of *A. remota* (MEAR).

**Methods:**

The MEAR was administered at doses of 200, 400, 600 and 800 mg/kg to four groups of mice (six animals per group) orally in castor oil diarrhea model. The effect of the extract on enteropooling and gastrointestinal transit model was also evaluated using the same grouping and dosing. Two other groups, one as control and the other as standard (loperamide 5 mg/kg) were used for comparison with the treatment groups.

**Results:**

The extract at the doses of 400, 600, and 800 mg/kg produced a dose-dependent and significant inhibition both on the frequency and onset of diarrhea. The percentage purging frequency was 53.4, 66.7, 79.6, and 66.7 % (*p* < 0.001) at three doses of MEAR (400, 600, and 800 mg/kg) and with loperamide (5 mg/kg), respectively. The percentage inhibition in intestinal fluid accumulation was 42.5, 62.1, and 74.2 % (*p* < 0.001) at the doses of 400, 600 and 800 mg/kg of MEAR, respectively. The MEAR also inhibited the intestinal transit of charcoal meal in a dose dependent manner both in the normal and castor oil induced intestinal transit.

**Conclusion:**

This study has shown that the 80 % methanol extract of *A. remota* contains pharmacologically active substances with significant antimotility and antisecretory effect contributing for its antidiarrheal activity.

## Background

Plant extracts are some of the most attractive sources of new drugs and have shown to produce promising results in the treatment of diarrhea [1]. There is a continuous and urgent need to discover new antidiarrheal compounds with diverse chemical structures and novel mechanisms of action. Therefore, researchers are increasingly turning their attention to folk medicine, looking for new leads to develop better drugs against diarrheal diseases [2, 3].

*Ajuga remota* is an erect rhizomatous pubescent herb that belongs to the genus *Ajuga*, found growing in the grasslands and other geographic parts of East Africa especially in Kenya and Ethiopia [4]. In Ethiopia, one of the vernacular name of *A. remota* is Armagusa (oromiffa), the name given by the community that uses this plant for the management of diarrhea [5]. The herb is not eaten by animals, birds or insects. This is probably due to the very bitter taste of almost all its parts [4]. The species *A. remota* is also known by other names such as *A. integrifolia Buch.-Ham,* and *A. bracteosa Wall.ex.Benth* [6].

Several studies are conducted on many species of the genus *Ajuga* and their active compounds have been identified. These efforts have led to the isolation of a number of compounds, including phytoecdysteroids [7], neo-clerodane diterpenes, diterpenoids (ajugarin I, II, III, IV &V, and others) [8], triterpenes, specific-sterols like beta-sitosterol, gamma-sitosterol, ceryl alcohol, anthocyanidin-glucosides, iridoid glycosides [4, 9], flavonol glycosides [4], quinolones, withanolides, flavonoids, tannins, triglycerides and essential oils [10].

In East Africa, plants of the genus *Ajuga* have been used as a remedy for fever, toothache, severe stomachache, dysentery, high blood pressure, malaria, edema, pneumonia and liver problems [4, 10]. In North Africa, plants of the genus *Ajuga* are used to treat diabetes and hypertension, as a panacea (cure-all), specifically for gastrointestinal disorders, and as an anthelmintic [10]. In traditional Chinese pharmacopoeia, plants of the genus *Ajuga* are known to produce a diuretic effect [10, 11]. Other ethnobotanical claims of the plant include treatment of diarrhea, gout, jaundice, amenorrhea, yellow fever, and as antiinfective [4, 5, 12].

Pharmacological studies have been carried out with *A. remota* since 1976 and it has been reported that *A. remota* possesses antimalarial activity [4], analgesic activity [13], anti-Human Immunodeficiency Virus Type 1 (HIV-1) and Type 2 (HIV-2) activity [14], antioxidant /oxygen scavenging activity [15], diuretic activity [11], antibacterial activity [16] as well as anthelmintic activity [14]. There are different mechanisms by which *A. remota* may have antidiarrheal effect. Some of the possible mechanisms are through its anthelmintic and antibacterial activities. The published reports on traditional use of *A. remota* for the treatment of diarrhea [5, 12] and the reported anthelmintic and antibacterial activity studies [14, 16] clearly warrant further scientific studies on antidiarrheal effect. Therefore, the present study aimed to evaluate whether the antidiarrheal effect also include antimotility and antisecretory effect using 80 % methanol extract of *A. remota*.

## Methods

### Chemicals

The chemicals used in the experiment include distilled water (Ethiopian Pharmaceutical Manufacturing, Ethiopia), loperamide HCl (Daehwa Pharmaceutical, Republic of Korea), castor oil (Remkaln General Trading P.LC., Amman Pharmaceutical Industries Co., Jordan), methanol (CARLO ERBA reagents, Germany), tween 80 (Atlas Chemical Industries Inc., UK), activated charcoal (India), chlorform (Finkem Laboratory Reagent, India), atropine sulphate inj.(0.1 %) (Jeil Pharm.Co.,Ltd., Korea).

### Plant material

The aerial parts of the plant *A. remota* were collected from a place called Karakore, which is located in Kolfe Keraniyo sub-city, Woreda 02 near to Medhanealem church in western part of Addis Ababa in December 2013. Identification and authentication of the plant specimen was done by a taxonomist at the National Herbarium, College of Natural and Computational Sciences, Addis Ababa University, where a voucher specimen (Voucher Specimen number TY001) has been deposited for future reference.

### Experimental animal

Swiss albino mice of either sex bred in the animal house unit of School of Pharmacy and having weights ranging from 20 to 30 g were used for the experiment. The mice were housed in cages, consisting of 6–10 mice per cage, under standard environmental conditions (a 12 h/12 h light/dark cycle); they were acclimatized for a period of 7 days before beginning the actual experiment and were provided with the standard laboratory pellet and tap water *ad libitum*. The care and handling of mice were in accordance with internationally accepted guidelines for use of animals [17] and were approved by Research and Ethics committee of School of Pharmacy, Addis Ababa University.

### Extraction of the plant material

After collection, the aerial parts of the plant were cleaned to remove dust and dirt then sliced to smaller pieces and dried at room temperature under shade for more than 3 weeks. The dried and sliced pieces were then powdered finely using mortar and pestle and subjected to extraction.

### Preparation of 80 % methanol extract

Maceration technique was used for the extraction of plant material using 80 % methanol. One hundred gram of the aerial parts of the powdered plant material was subjected to maceration process with about 500 ml of 80 % methanol at room temperature for 72 h while shaking occasionally. The extract was then filtered through a muslin cloth and Whatman No.1 filter paper and the marc was remacerated twice using the same volume of solvent to exhaustively extract the plant material. The methanol was then removed from the extract by evaporation under reduced pressure using a rota vapor (BUCHI Rota vapour R-200, Switzerland) at 40 °C. The extract was then dried using a lyophilizer to remove the remaining water. The resulting dry hydroalcoholic extract was weighed and calculated for percentage yield, which was 15.5 % w/w. The dried plant extract was reconstituted with 2 % tween 80 for oral administration.

## Experimental design

### Grouping and dosing

Animals were randomly assigned into six groups each consisting of six mice of either sex (weighing 20–30 g) and were fasted for 18 h before the test with free access to water for antidiarrheal test. Negative controls were treated with the vehicle used for reconstitution (10 ml/kg), 2 % tween 80 v/v. Positive controls were treated with standard drug, loperamide HCl (5 mg/kg) or atropine sulphate (3 mg/kg) depending on the type of the model used. Four treatment groups were treated with four different doses of MEAR; 200, 400, 600 and 800 mg/kg orally by using oral gavage. Dose selection was made based on the pilot test performed prior to commencement of the actual experiment.

### Antidiarrheal activity

#### Castor oil induced diarrhea model

The experiment was carried out according to the method described by Yadav and Tangpu [18]. After 1 h of treatment with extract, 2 % tween 80 or standard drug, diarrhea was induced by administration of 0.5 ml of castor oil orally to each mouse. The mice were then housed individually in transparent metabolic cages, the bottom of which was lined with white sheet of paper for observation of the number and consistency of fecal droppings. The papers were changed every hour to make the fecal droppings visible for counting and to check stool consistency. During observation period of 4 h, the onset of diarrhea, the number and weight of both dry and wet stools excreted by the animals were recorded and compared with the control for assessing the antidiarrheal activity. The onset was measured as the time interval in minutes between the administration of castor oil and the appearance of the first diarrheal stool. The total number of diarrheal feces of the control group was considered 100 %. Percent inhibition (PI) was calculated as follows;

$$ PI = \frac{Mean\  number\  of\  wet\  stools\  of\ \left( control\  group- treated\  group\right)}{Mean\  number\  of\  wet\  stools\  of\  control\  group}\times 100 $$

#### Castor oil-induced enteropooling

Intraluminal fluid accumulation was determined using the method of Maxwell [19], with slight modifications. After 1 h of treatment with extract, 2 % tween 80 (v/v) or standard drug, 0.5 ml castor oil was administered orally to each mouse to induce diarrhea. One hour after castor oil treatment, all mice were sacrificed by cervical dislocation, and the small intestine was ligated both at the pyloric sphincter and at the ileocecal junctions and dissected out. The small intestine was weighed. The intestinal contents were collected by milking into a graduated tube and the volume was measured. The intestines were reweighed and the differences between full and empty intestines were calculated.

#### Normal gastrointestinal transit in mice

Normal gastrointestinal transit was investigated in mice according to the methods described by Bakare et.al and Shakuntala et.al [20, 21]. One hour after the administration of 2 % tween 80 (v/v), extract or standard drug, each animal was given 1 ml standard charcoal meal (10 % activated charcoal suspension in 2 % tween 80). The mice were then sacrificed 1 h after the administration of the charcoal meal, the abdomen were opened and the small intestine was immediately isolated. The length of the intestine from pylorus to the caecum (LSI) and the distance traveled by the charcoal (LM) were measured. The peristaltic index (PI) for each mouse was calculated, expressed as percentage of the distance traveled by the charcoal meal relative to the total length of the small intestine. The percentage inhibition relative to the control was also calculated as:

$$ PI = LM/LSI\ x\ 100\% $$

Where: PI = Peristaltic Index

LM = Length of Charcoal Meal; LSI = Length of Small Intestine

$$ \%\  Inhibition = \frac{Mean\  of\  distance\  travelled\ by\  marker\  of\ \left( Control\ \hbox{--}\ Test\right)\  group}{Mean\  of\  distance\  travelled\ by\  marker\  of\  control\  group}\times 100 $$

#### Castor oil induced gastrointestinal transit in mice

This experiment was carried out using the method described by Al-Taher [22]. One hour after the administration of 2 % tween 80 v/v, extract or standard drug, castor oil (0.5 ml/mouse) was administered orally to these animals to induce diarrhea. After 1 h of castor oil administration, all animals received 1 ml of charcoal meal marker (10 % charcoal suspension in 2 % tween 80) orally. All the animals were sacrificed after 1 h of marker administration and the small intestine was rapidly dissected out and placed on a clean white sheet of paper that was stretched on top surface of the table. The intestine was carefully inspected and the distance travelled by charcoal meal plug from the pylorus to caecum was measured and expressed as a percentage of the total distance of the small intestine. The length of the whole intestine was also measured.

### In vivo antidiarrheal index (ADI)

The in vivo antidiarrheal index (ADI in vivo) was then expressed according to the formula developed by Aye-Than as described [23].

$$ \mathbf{A}\mathbf{D}\mathbf{I}\;\boldsymbol{in}\ \boldsymbol{vivo}=\sqrt[\mathbf{3}]{\ }\left(\mathbf{D}\ \mathbf{freq} \times \mathbf{G}\ \mathbf{m}\mathbf{e}\mathbf{q} \times \mathbf{P}\ \mathbf{freq}\right) $$

where, **D**_**freq**_ is the delay in defecation time or diarrheal onset (as % of control),

**G**_**meq**_ is the gut meal travel reduction (as % of control), and

**P**_**freq**_ is the purging frequency, or reduction in the number of stools (as % of control).

### Phytochemical analysis

Phytochemical tests were carried out for the methanol extract of the plant using standard procedures [24, 25] to identify the presence of secondary metabolites, including alkaloids, flavonoids, terpenoids, tannins, phenolics, anthraquinones, glycosides, steroids, and saponins.

### Statistical analysis

The results were expressed as mean ± SEM and they were analyzed statistically using One way ANOVA followed by post-hoc tukey test to find out significance difference between control group against each test groups separately. The value of *P* < 0.05 was considered statistically significant. Dose-dependent effect was confirmed using linear regression analysis. Data were tested for a normal distribution using Shapiro-Wilk’s normality test.

## Results

### Castor oil-induced diarrhea

Pretreatment of mice at the doses of 400, 600, and 800 mg/kg of MEAR caused a concentration-dependent and significant decrease in the frequency of stooling (Regression coefficient; *R*^2^ = 1, for both), decrease in the weight of wet stools (*R*^2^ = 0.963), and delay in the onset of diarrhea (*R*^2^ = 0.995). The percentage purging frequency relative to controls was 53.7, 66.7, 79.6, and 66.7 % (*p* < 0.001) at three doses of MEAR (400, 600, and 800 mg/kg) and with loperamide (5 mg/kg), respectively as shown in Table 1. The percentages relative to controls for diarrhea onset were 93.7 (*p* < 0.01), 145.7, and 183.5 % (*p* < 0.001) at the doses of 400, 600, and 800 mg/kg of MEAR, while with loperamide (5 mg/kg) this value was 137.3 %(*p* < 0.001) compared to controls as shown in Table 1. The extract at the dose of 200 mg/kg had no significant effect whereas at the dose of 800 mg/kg produced maximal inhibitory effect on all the diarrhea parameters measured; similar results were obtained for all the other models tested below.

**Table 1:** Effect of the 80 % methanol extract of *A. remota* on castor oil induced diarrhea in mice

Group (mg/kg)	Onset of diarrhea (min)	Mean wt. of wet stools in 4 h (g)	Mean no. of wet stools in 4 h	Total no. of stools in 4 h	PI
Control	67.00 ± 2.51	0.69 ± .04	9.00 ± 1.09	11.00 ± 1.26	
Loperamide5	174.33 ± 14.99^a***^	0.22 ± .05^a***^	3.00 ± .73 ^a***^	4.00 ± .07 ^a***^	66.7 %
MEAR 200	94.67 ± 1.64^b**c***d**^	0.53 ± .02^b***c***d**^	6.83 ± .60^b**c***d**^	10.00 ± .57^b**c***d**^	12.9 %
MEAR 400	151.33 ± 1.42^a***f*^	0.35 ± .01^a***^	4.17 ± .30^a***c*^	6.00 ± .73^a**f**^	53.7 %
MEAR 600	164.67 ± 15.14^a***^	0.28 ± .05^a***^	3.00 ± .63 ^a***^	4.00 ± .85^a***^	66.7 %
MEAR 800	190.00 ± 19.65 ^a***^	0.15 ± .05^a***e*^	1.83 ± .60^a***^	2.00 ± .94 ^a***^	79.6 %

Values are expressed as Mean ± SEM.(*n* = 6 mice) ^a^against control, ^b^against standard, ^c^against 800 MEAR, ^d^against 600 MEAR, ^e^against 400 MEAR, ^f^against 200 MEAR ^*^ *p* < 0.05, ^**^ *p* < 0.01, ^***^ *p* < 0.001., numbers refers to dose in mg/kg, PI refers to percent inhibition of defecation

### Castor oil induced enteropooling

**Fig. 1:**
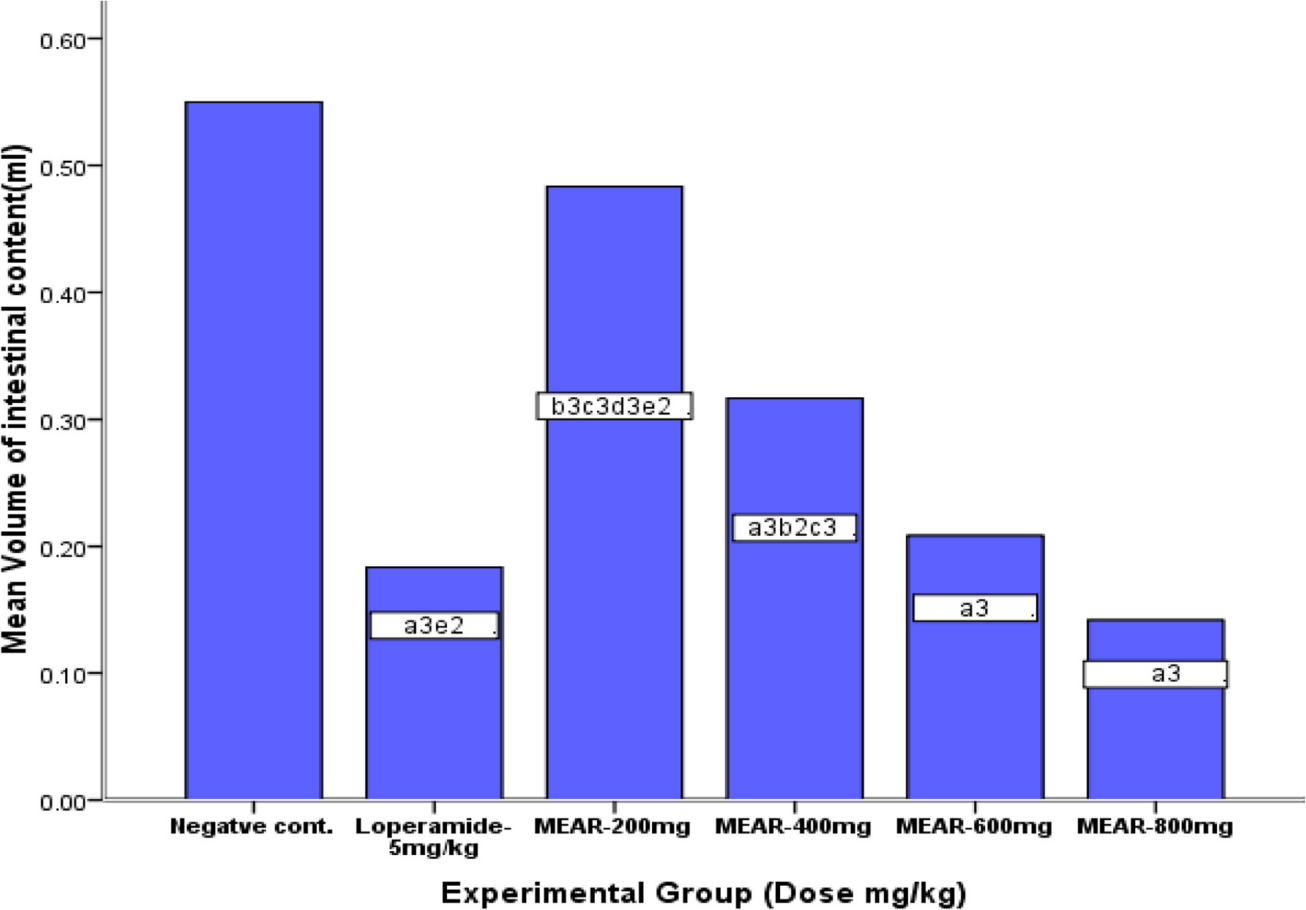
Effects of MEAR and controls on castor oil-induced enteropooling (volume of intestinal content) in mice (*n* = 6). Data are mean ± SEM. ^a^against control, ^b^against standard, ^c^against 800 MEAR, ^d^against 600 MEAR, ^e^against 400 MEAR, ^1^ *p* < 0.05, ^2^ *p* < 0.01, ^3^ *p* < 0.001. MEAR refers to 80 % methanol extract of *Ajuga remota*, control: groups treated with 10 ml/kg 2 % tween 80

**Fig. 2:**
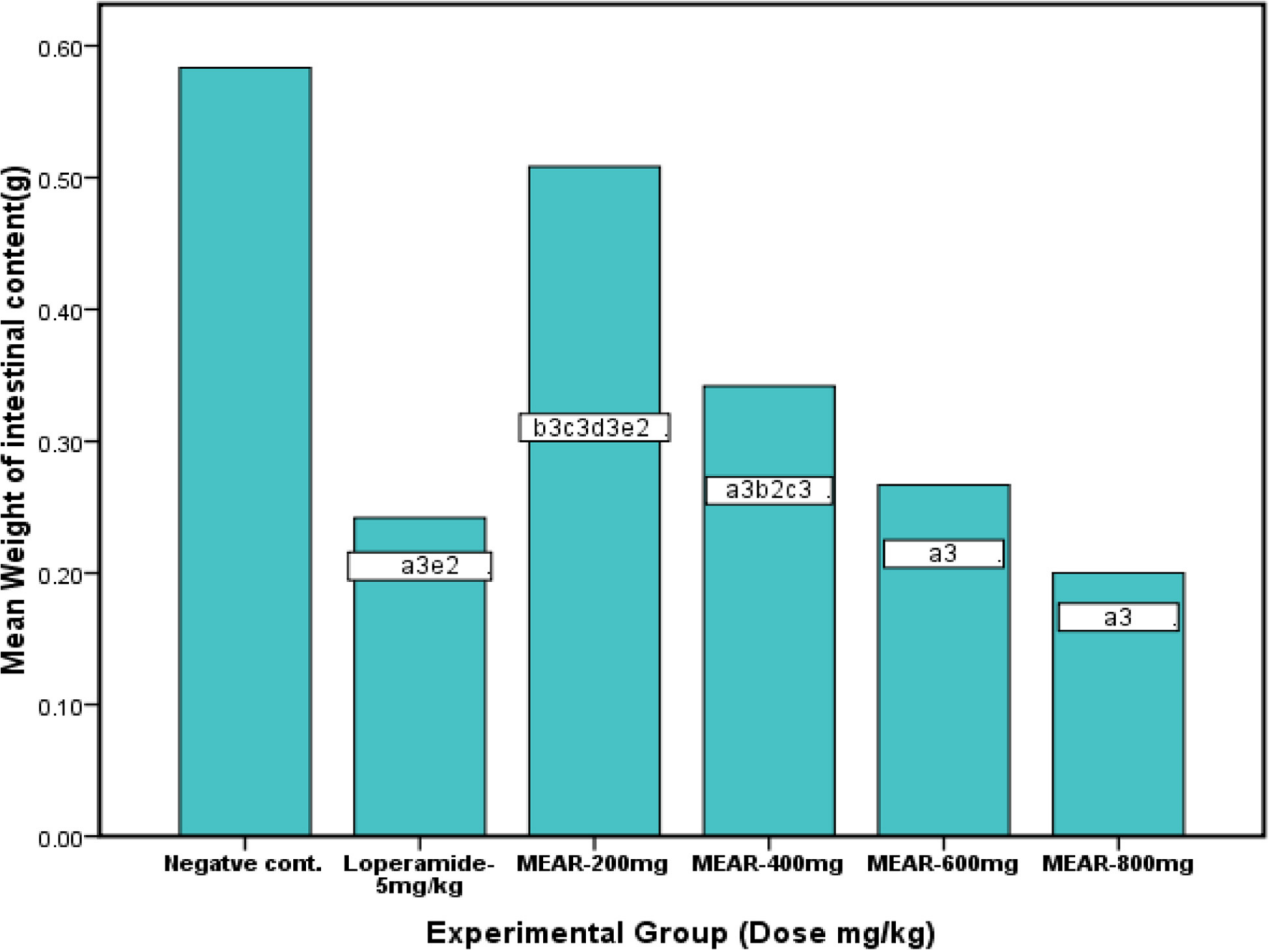
Effects of MEAR and controls on castor oil-induced enteropooling (weight of intestinal content) in mice (*n* = 6). Data are mean ± SEM. ^a^against control, ^b^against standard, ^c^against 800 MEAR, ^d^against 600 MEAR, ^e^against 400 MEAR, ^1^ *p* < 0.05, ^2^ *p* < 0.01, ^3^ *p* < 0.001. MEAR refers to 80 % methanol extract of *Ajuga remota*, control: groups treated with 10 ml/kg 2 % tween 80

Percentage inhibition in intestinal fluid accumulation was 42.4, 62.1, and 74.2 % (*p* < 0.001) at the doses of 400, 600 and 800 mg/kg of MEAR, respectively, and with loperamide HCl (5 mg/kg) this value was 66.6 % (*p* < 0.001) as shown in Fig. 1. The extract showed a dose-dependent inhibition (*R*^2^ = 0.981) in castor oil induced intestinal fluid accumulation. Similar results were obtained for the weight of intestinal content at the different doses of the extract, compared to the control group as shown in Fig. 2.

### Normal gastrointestinal propulsion in mice

The MEAR inhibited the normal intestinal transit of charcoal meal plug by 35.4, 49.1, and 63.9 % *(p < 0.001)* at the doses of 400, 600 and 800 mg/kg, respectively, while atropine sulphate at the dose of 3 mg/kg showed 55.0 % (*p < 0.001)* inhibition as shown in Table 2. The extract inhibited significantly the normal intestinal propulsion in a dose dependent manner (*R*^2^ = 1.000).

**Table 2:** Normal gastrointestinal transit in mice treated with the 80 % methanol extract of *A. remota*

Group (mg/kg)	Mean of the total length of intestine (cm)	Mean of distance traveled by charcoal meal (cm)	Peristaltic index (PI)	(%) Inhibition
Control	57.77 ± 1.09	40.74 ± 1.25	70.51	0.0
Atropine 3	55.96 ± 1.28	18.33 ± 0.56 ^a***e*^	32.76	55.0
MEAR 200	55.71 ± 1.11	37.56 ± 1.51^b***c***d***e***^	67.43	7.7
MEAR 400	53.58 ± 0.93	26.28 ± 2.15^a***b*^	49.05	35.5
MEAR 600	54.47 ± 1.17	20.70 ± 1.18^a***^	38.00	49.2
MEAR 800	55.30 ± 1.21	14.69 ± 0.55^a***e***^	26.58	63.9

Values are expressed as mean ± SEM.(*n* = 6 mice) ^a^against control, ^b^against standard, ^c^against 800 MEAR, ^d^against 600 MEAR, ^e^against 400 MEAR; ^*^ *P* < 0.05, ^**^ *p* < 0.001, ^***^ *p* < 0.001. 200, 400, 600 and 800 mg/kg represent doses of MEAR; Control: control group taking 10 ml/kg 2 % tween 80, Atropine : Atropine sulphate 3 mg/kg

### Castor oil induced gastrointestinal transit in mice

The MEAR inhibited the intestinal transit of charcoal meal induced by castor oil by 33.3, 53.8 and 64.6 % *(p < 0.001)* at the doses of 400, 600 and 800 mg/kg, respectively, while atropine sulphate at the dose of 3 mg/kg showed 61.4 % (*p < 0.001)* inhibition. The extract inhibited significantly in castor oil induced intestinal transit in a dose dependent manner (*R*^2^ = 1.000).

### In vivo *antidiarraheal index*

The antidiarrheal index (ADI) is a measure of the combined effects of these different components of diarrhea such as purging frequency, onset of diarrheal stools, and intestinal fluid accumulation. Results for the in vivo *antidiarraheal indices* were 16.3, 60.8, 80.5 and 98.1 % at the doses of 200, 400, 600 and 800 mg/kg, p.o. of MEAR respectively, while the 800 mg/kg of the extract gave a maximum index of 98.1 %. The extract produced antidiarrheal activity as shown by the antidiarrheal index in a dose dependent manner (*R*^2^ = 1.000).

### Phytochemical analysis

The phytochemical analysis of MEAR tested positive for alkaloids, flavonoids, terpenoids, tannins, phenolics, anthraquinones, glycosides, steroids, and saponins.

## Discussion

This study was carried out to evaluate the antidiarrheal activity of the plant extract of *A. remota* in experimental animals, and determine the possible mechanism of action. We have evaluated the acclaimed properties of the plant using different animal models. Relevantly, the 80 % methanol extract of the aerial parts of *A. remota* showed a statistically significant and dose-dependent inhibition in all diarrheal parameters: onset of diarrhea, weight of wet stools, number of wet stools, and total number of stools as compared to the control group. This result is in accordance with previous claims in respect of antidiarrheal herbs. Antidiarrheal plants are known to reduce number of wet stools, consistency of fecal droppings as well as delay in the onset of diarrhea as reported for *Pterocarpus erinaceus* [19]*, Saussurea lappa*, *Lithocarpus dealbata and Urena lobata* [18].

Castor oil was used in this study to induce diarrhea since castor oil model, incorporates both secretory and motility diarrhea [26–28]. Castor oil is composed principally of ricinoleic acid [29]. The ricinoleic acid readily forms ricinoleate salts with Na^+^ and K^+^ in the lumen of the intestine. Ricinoleate has several actions that could account for its anti-absorptive effect on the mucosa. It inhibits the enzyme Na^+^ − K^+^ ATPase and increases the permeability of the intestinal epithelium [30, 31]. Thus; one possible anti-diarrheal activity of the extract against castor oil induced diarrhea may be attributed to its anti-electrolyte permeability action [30]. Ricinoleic acid also causes local irritation and inflammation of the intestinal mucosa leading to prostaglandin (PG) release, which causes an increase in the net secretion of water and electrolytes into the small intestine [30, 32]. Inhibitors of prostaglandin biosynthesis delayed castor oil induced diarrhea [33]. Based on these facts, it is reasonable to suggest that the extract may reduce PG induced secretion of water and electrolytes due to the inhibition of prostaglandin synthesis [34]. Investigations of the mode of action indicate that tannins and flavonoids present in the plant may increase colonic water and electrolyte reabsorption [3]. Thus, in general the antidiarrheal activity of the extract against experimentally induced diarrhea by castor oil may be attributed to an increase in fluid and electrolytes absorption and/or to a slowing down of intestinal transit, allowing more time for absorption to occur since castor oil induces acceleration of the intestinal transit because of its motor activities and prevent absorption of water and solutes in the lower intestine [34].

Studies on castor oil - induced intestinal fluid accumulation showed that the extract reduced both the weight and volume of intraluminal contents. These effects, which are direct consequences of reduced water and electrolytes secretion into the small intestine, suggest that the extract may enhance electrolyte absorption from the intestinal lumen consistent with inhibition of hypersecretion [35]. Again it is likely that the enhanced electrolyte absorption by the extract may have encouraged the absorption of other intestinal solute contents like nutrients that in turn may have created an osmotic gradient across enterocytes which stimulated water absorption [35]. Thus, another possible antidiarrheal activity of the extract could be the reduction in net fluid and solutes secretion, since a great part of the laxative effect of castor oil is related to increase in fluid secretion [34] and this fluid content is the principal determinant of stool volume and consistency [30].

Tannins present in plant, denature proteins in the intestinal mucosa forming protein tannate complex. The complex forms a coat over the intestinal mucosa and makes the intestinal mucosa more resistant to chemical alteration and reduces secretion [10, 36]. In addition, flavonoids present antioxidant properties which are presumed to be responsible for the inhibitory effects exerted upon several enzymes including those involved in the arachidonic acid metabolism [37], thus, reducing prostaglandin induced fluid secretion. Furthermore, a previous study has also shown that the plant possesses anti-oxidant activity [15].

The 80 % methanol extract of *A. remota* inhibited gastrointestinal propulsion in both the normal or castor oil induced intestinal transit. This finding suggests that the extract acts on all parts of the intestine. A decrease in the motility of gut muscles increases the stay of substances in the intestine. This allows better water and electrolyte absorption. This is indicative of the ability of the plant to alter normal peristaltic movement and hence decrease the movement of materials in the intestinal tract allowing greater time for absorption [21, 26]. MEAR changed normal intestinal transit in mice at doses showing antidiarrheal activity. Since the extract shows antipropulsive effect on normal intestine similar to atropine it is likely that the antidiarrheal effect could be due to anticholinergic activity.

Investigations on the mode of action indicate that phytochemicals like phenolics, alkaloids and terpenoids act by inhibiting intestinal motility [3]. Apart from this, flavonoids have been ascribed to their ability to inhibit intestinal motility and electrolytic secretion [3], as well as contractions induced by spasmogenics [34]. Studies on the functional role of tannins also revealed that they can also reduce the peristaltic movements and intestinal secretions by reducing the intracellular Ca^2+^inward current or by activation of the calcium pumping system (which induces the muscle relaxation) [22] attributed by spasmolytic and calcium channel blocking (CCB) activities of tannins present in the plant extract [38]. Sesquiterpenes, diterpenes, terpenes, and other terpenoid derivatives are known for inhibiting release of autocoids and prostaglandins, thereby inhibit the motility and secretion induced by castor oil [39].

Hypermotility characterizes forms of diarrhea where the secretory component is not the causative factor [35]. Pre-treatment with the extract suppressed the propulsive movement of charcoal meal plug through the gastrointestinal tract in castor oil induced gut transit which clearly indicates that the aerial parts of the plant extract may be capable of reducing the frequency of stooling in diarrheal conditions. Delay in gastric motility causes further absorption of water from feces and may additionally contribute to reducing its watery texture. From the results of this study, it is likely that the extract inhibits gastrointestinal hypermotility in diarrhea through anticholinergic alkaloids present in *A.remota* [35, 38].

The antidiarrheal index (ADI) is a measure of the combined effects of these different components of diarrhea such as purging frequency, onset of diarrheal stools, and intestinal fluid accumulation [23]. The 80 % methanol extract of the aerial parts of *A. remota* produced a dose-dependent antidiarrheal index which implies that the plant extract produces antidiarrheal activity in a dose-related manner. The plant has also been shown to possess antibacterial, antiviral, antifungal, and anthelmintic properties [10, 26]. Such activities of the plant could account additional benefit providing a wider cover for its use in diarrhea of different etiologies including those of infectious diarrheas and thus can serve as a profitable alternative for treating such diarrheas [10, 26]. This study has been undertaken by using a crude hydroalcoholic extract, and that the different biological activities assayed herein, may not be due to a single constituent since the crude extracts contain several compounds acting on different mechanisms. In addition, the interplay of the constituents in the crude extract may result in better activity due to synergism or may lead to a decrease in toxicity and it is possible that pure compounds may not necessarily behave in the same manner as the natural extracts [3, 24].

## Conclusion

This study has shown that the 80 % methanol extract of *A. remota* contains pharmacologically active substances with significant antimotility and antisecretory effect contributing for its antidiarrheal activity in all experimental models used in the study. These attributes may provide the rationale for the use of *A. remota* for treating diarrhea and hence, on a preliminary basis it can be claimed as a potential therapeutic option in the effective management of diarrhea, thus supporting its folkloric use by traditional healers for this purpose.

## Abbreviations

*A. bracteosa*: *Ajuga bracteosa*
*A. integrifolia*: *Ajuga integrifolia*
*A. remota*: *Ajuga remota*
ADI: Antidiarrheal index
ANOVA: Analysis of variance
CCB: Calcium channel blockers
HIV: Human immunodeficiency virus
LM: The distance traveled by the charcoal meal
LSI: Length of the intestine from pylorus to the caecum
MEAR: Methanol extract of *Ajuga remota*
PG: Prostaglandin
PI: Peristaltic index
PO: Per os
